# Ultrasound findings for the diagnosis of biliary atresia in neonates

**DOI:** 10.1590/0100-3984.2024.0102

**Published:** 2025-04-25

**Authors:** Elazir Barbosa Mota Di Puglia, Pedro Augusto Nascimento Daltro, Heron Werner Junior, Miriam Menna Barreto, Flávia Angélica Ferreira Francisco, Sérgio Ferreira Alves Junior, Ivonete Siviero, Claudia Renata S. Paio Rezende, Edson Marchiori

**Affiliations:** 1 Universidade Federal do Rio de Janeiro (UFRJ), Rio de Janeiro, RJ, Brazil; 2 Alta Diagnósticos (DASA), Rio de Janeiro, RJ, Brazil; 3 Instituto Fernandes Figueira (IFF/Fiocruz), Rio de Janeiro, RJ, Brazil; 4 Image Kids Ultrassonografia, Petrópolis, RJ, Brazil

**Keywords:** Diagnosis, Abdomen/physiopathology, Gallbladder/physiopathology, Ultrasonography/methods, Biliary atresia/diagnostic imaging, Pediatrics, Diagnóstico, Abdome/fisiopatologia, Vesícula biliar/fisiopatologia, Ultrassonografia/métodos, Atresia biliar/diagnóstico por imagem, Pediatria

## Abstract

**Objective:**

To investigate and identify the main abdominal ultrasound findings in patients with biliary atresia (BA).

**Materials and Methods:**

This was a retrospective study of the ultrasound images of 44 patients with neonatal cholestasis. We excluded 18 patients in whom a final diagnosis of BA was not confirmed or who were lost to clinical follow-up. The main ultrasound findings evaluated were gallbladder length and morphology; triangular cord thickness; hepatic artery enlargement; hepatic subcapsular flow; cysts in the porta hepatis; presence of a distinct triangular cord with linear, tubular, or round hypoechoic portions; and polysplenia syndrome.

**Results:**

Abnormal gallbladder morphology and triangular cord thickening were the main ultrasound findings in the patients with BA. Gallbladder abnormalities were present in all patients. Hepatic artery enlargement was the third most common finding, present in 19 (73%) patients. Six patients (23%) had subcapsular arterial flow and four (15%) had cysts in the porta hepatis. Hypoechoic or cystic portions of the triangular cord were present in three patients (11%), and we found that BA was accompanied by polysplenia syndrome in three patients (11%).

**Conclusion:**

Ultrasound is the examination of greatest diagnostic relevance in the investigation of cholestasis in newborns and infants; it enables the establishment of BA suspicion and the indication for laparotomy with intraoperative cholangiography.

## Abstract (pt)

**Objetivo:**

Investigar e analisar os principais achados de imagem no ultrassom de abdome de pacientes com atresia de vias biliares.

**Materiais e Métodos:**

Estudo retrospectivo de 44 pacientes com quadro de colestase neonatal. Foram excluídos do estudo 18 pacientes que não tiveram diagnóstico final de atresia de vias biliares confirmado ou que se perderam acompanhamento clínico. Os principais achados no ultrassom foram: eixo longitudinal e morfologia da vesícula biliar, caracterizados pela ausência da mucosa hiperecogênica e seus contornos irregulares; cordão hiperecogênico; artéria hepática calibrosa; fluxo arterial subcapsular; cisto no porta hepatis; imagens lineares e hipoecoicas no interior do cordão hiperecogênico; e síndrome da poliesplenia.

**Resultados:**

Morfologia da vesícula biliar esteve presente em todos os pacientes estudados. Os principais achados no ultrassom foram alterações morfológicas na vesícula biliar e cordão hiperecogênico. Artéria hepática calibrosa foi o terceiro achado mais frequente, presente em 19 pacientes (73%), seguido de fluxo arterial subcapsular em seis (23%), cisto no porta hepatis em quatro (15%), imagens hipoecoicas arredondadas ou lineares no interior do cordão em três (11%) e síndrome da poliesplenia em três pacientes (11%).

**Conclusão:**

O ultrassom é o método diagnóstico de escolha na avaliação dos pacientes com colestase suspeitos para atresia de vias biliares, permitindo a suspeição e a indicação correta da laparotomia com colangiografia intraoperatória.

## INTRODUCTION

Neonatal jaundice has many causes. Most cholestatic conditions can be classified as obstructive or hepatocellular in origin. Biliary atresia (BA) accounts for more than 90% of all cases of obstructive cholestasis^(1^, ^2^, ^3)^. Hepatocellular cholestasis results from the impairment of bile formation and indicates the defective functioning of most or all hepatocytes. The majority of hepatocellular cholestasis cases are idiopathic neonatal hepatitis^(1^, ^4)^.

Categorized as a chronic liver disease, BA is characterized by inflammation and destruction of the bile ducts, leading to progressive fibrosis of the extrahepatic and, in many cases, intrahepatic bile ducts. When left untreated, it progresses to cirrhosis, portal hypertension, liver failure, and death within two years^(1^, ^5^, ^6)^. It is a disease exclusive to childhood, with no significant sex predilection ^(4^, ^7)^. Most children with BA present with jaundice, acholia or fecal hypocholia, choluria, and varying degrees of hepatomegaly^(1^, ^4^, ^5^, ^6)^. It is the main cause of obstructive jaundice in childhood and the most common indication for liver transplantation in the pediatric population^(8^, ^9^, ^10)^.

The palliative correction of BA is performed with the Kasai procedure (portoenterostomy) to reestablish hepatic flow. Early, accurate preoperative diagnosis of BA is necessary because the Kasai procedure has been shown to be more successful when performed within the first 60 days of life, with the main factors for good prognosis being early diagnosis and surgical intervention^(11^, ^12^, ^13^, ^14)^. Ultrasound has played an important role in screening for infantile cholestasis, enabling the establishment of BA suspicion and the indication for laparotomy with intraoperative cholangiography^(15)^. Several ultrasound findings have been described as useful predictors of BA^(4^, ^8^, ^9^, ^10^, ^11^, ^10^, ^12)^. Atretic gallbladder^(8)^ and the triangular cord (TC) sign^(4^, ^9^, ^10^, ^16)^ have been shown to be useful indicators, with variable diagnostic performance.

The objective of this study was to evaluate the main ultrasound findings of BA. Whole-abdomen ultrasound scans of 26 patients with confirmed diagnoses of BA were analyzed.

## MATERIALS AND METHODS

The sample for this retrospective analysis was drawn from a total of 44 patients with neonatal cholestasis and suspected BA, who were referred to the Hepatology Department or Pediatric Surgery Department of the Instituto de Puericultura e Pediatria Martagão Gesteira (Martagão Gesteira Institute of Childcare and Pediatrics), in the city of Rio de Janeiro, Brazil, between January 2016 and March 2023. Patient ages ranged from 18 days to 195 days (mean, 77 days), and their symptoms were jaundice (conjugated hyperbilirubinemia), acholic stools, or both. The diagnosis of BA was made through intraoperative cholangiography and confirmed by histopathological analysis. Eighteen patients were excluded from the study because they had no confirmed BA diagnosis or were lost to outpatient follow-up.

All infants with suspected BA underwent abdominal ultrasound examinations, performed by a pediatric radiologist with 28 years of experience in pediatric examination. After the infants had fasted for 4–6 hours, the scans were performed with an Aplio 300 ultrasound system (Toshiba Medical Systems, Tokyo, Japan) with convex and linear multifrequency transducers at average frequencies of 6 MHz and 14 MHz, respectively. First, the presence and morphological characteristics of the gallbladder were assessed using the high-frequency linear transducer. Thereafter, the mothers were authorized to feed their infants during the examinations. Positivity was determined on the basis of the following: the lack of gallbladder visualization; and the detection of atresia, defined as a longitudinal gallbladder axis ≤ 1.5 cm, indistinct parietal mucosal line (undetectable throughout the entire gallbladder), with or without an irregular wall. The TC sign was recorded as present when hyperechoic thickening > 2 mm was detected anterior to the most distal portion of the right anterior branch of the portal vein or > 3 mm thickening was detected anterior to the portal vein bifurcation. The internal diameter of the proximal right hepatic artery (HA) was measured at the level of the right portal vein and values > 1.9 mm were considered positive for HA enlargement.

Hepatic subcapsular blood flow was assessed by color Doppler ultrasound, with the color box positioned on the subcapsular hepatic surface, near the falciform ligament. This finding was positive for BA when an arterial curve pattern was observed on the periphery of the liver parenchyma. The hepatic arterial waveform is pulsatile, with the peak corresponding to the peak systolic velocity and the trough corresponding to the end-diastolic velocity.

Positivity for cysts in the porta hepatis was recorded upon the detection of either macrocysts (> 5 mm) or microcysts (≤ 5 mm) anterior to the right branch of the portal vein in the porta hepatis. A distinct TC that contained linear, tubular, or round hypoechoic or cystic portions was taken to represent the cystic dilatations of the extrahepatic bile duct. Findings such as polysplenia, situs inversus, preduodenal portal vein, inferior vena cava anomalies, and other findings characterizing polysplenia syndrome were also recorded.

Secondary findings related to the later stages of BA (progression to hepatic fibrosis and portal hypertension), such as splenomegaly and hepatomegaly, were assessed via measurement of the oblique diameter of the right liver lobe and the splenic length of the spleen. The presence of ascites and collateral circulation were assessed with the linear transducer.

## RESULTS

**Figure 1:**
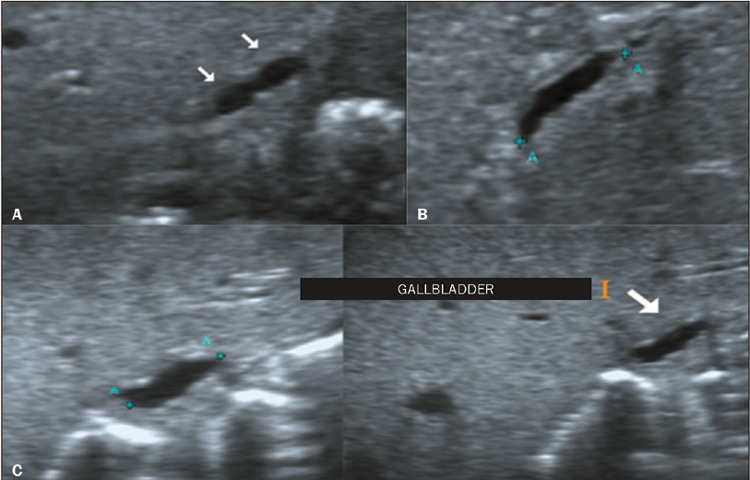
Cases of gallbladder atresia, as detected by ultrasound with a linear transducer, in infants with BA. **A:** A small gallbladder (arrow) with an irregular wall and abnormal shape. **B:** An abnormal gallbladder with wall irregularity that is more subtle. **C:** Another example of an atretic gallbladder.

**Figure 2:**
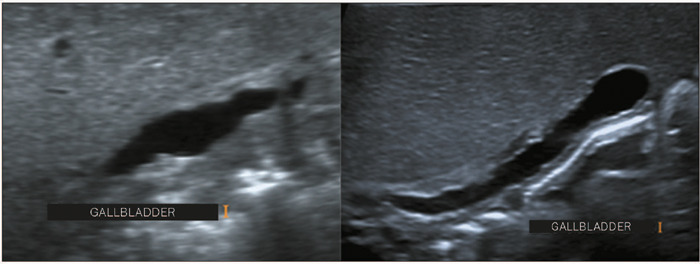
Ultrasound image obtained with a linear transducer of a gallbladder with a longitudinal axis > 1.5 cm, an indistinct mucosal line, with or without an irregular wall, in an infant with BA.

**Figure 3:**
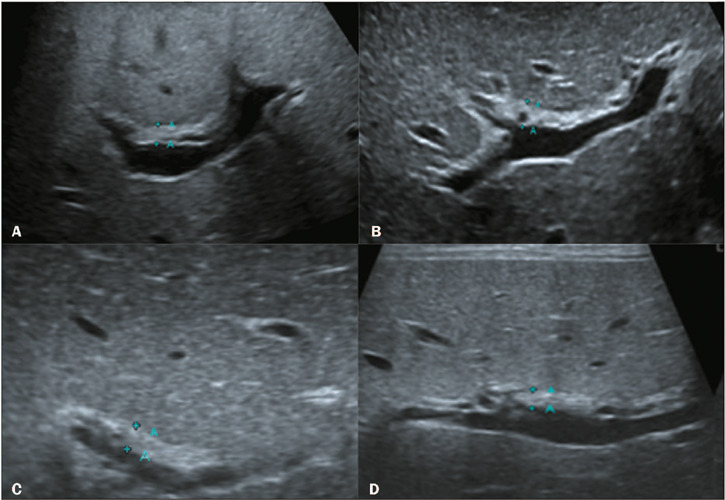
The TC sign, detected by ultrasound, in infants with BA. **A,B:** Images of a fibrotic TC with a thickness of 4 mm anterior to the bifurcation of the portal vein. **C,D:** The modified TC thickness of > 2 mm was a positive sign for the prediction of BA.

**Figure 4:**
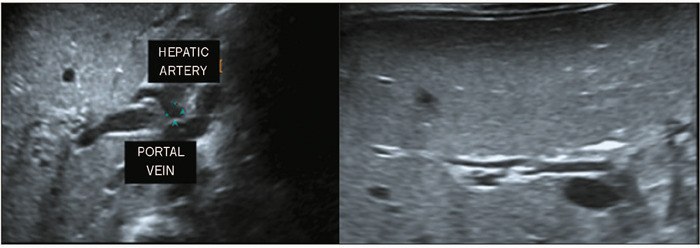
Oblique ultrasound images showing an enlarged HA (measuring 3 mm) in an infant with BA.

**Figure 5:**
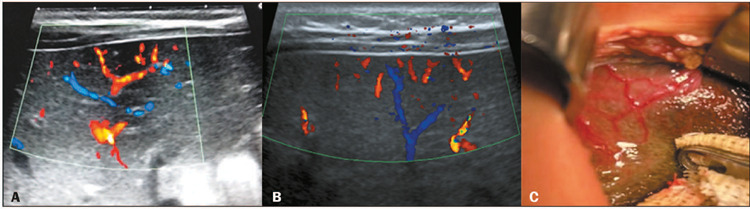
Color Doppler ultrasound images of abnormal arterial flow in infants with BA. **A,B:** Cases in which the hepatic arterial flow extended to the liver surface. **C:** Subcapsular telangiectasia was observed intraoperatively on the liver surface, corresponding to the subcapsular arterial flow detected by ultrasound.

**Figure 6:**
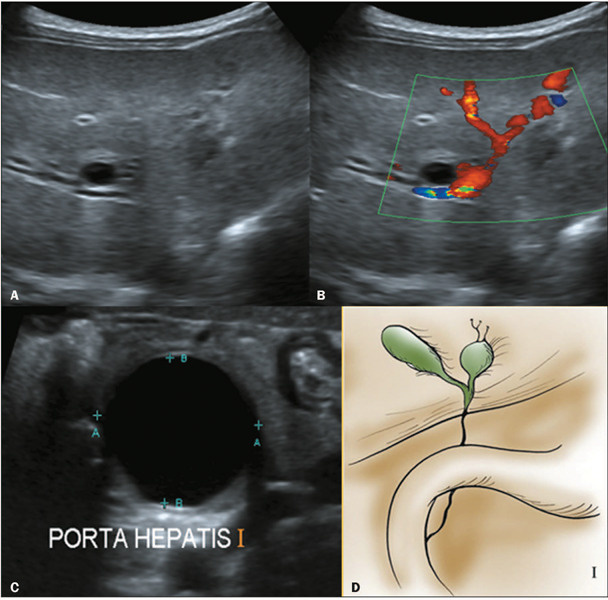
Abdominal ultrasound images showing a small cyst **(A,B)** and a macrocystic structure **(C)** in the porta hepatis. After surgery, the BA was confirmed to be subtype I **(D)** on the basis of the Japanese Society of Pediatric Surgeons criteria.

**Figure 7:**
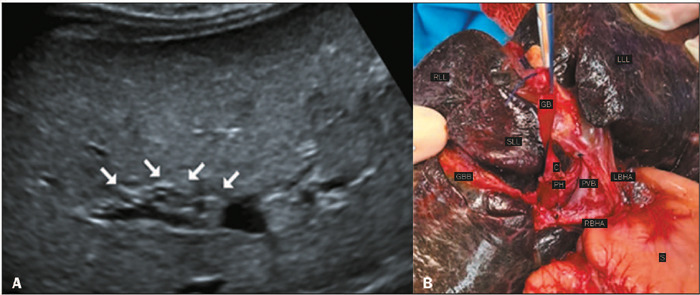
**A:** Abdominal ultrasound image showing the TC sign, with the cord having small hypoechoic portions (arrows). **B:** The remnants of the bile ducts were sectioned, and the Kasai procedure was performed. A cystic structure was observed in the porta hepatis.

**Figure 8:**
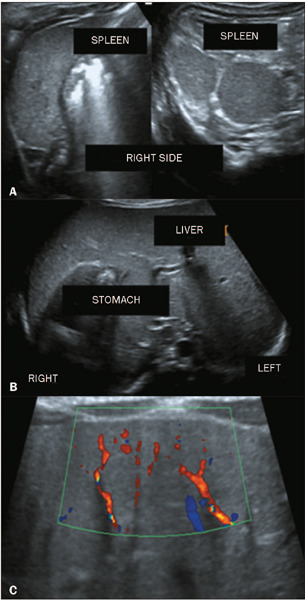
Transverse ultrasound images of a patient with polysplenia syndrome **(A),** small multiple spleens in the right upper quadrant **(B),** and situs inversus. The BA findings **(C)** included the TC sign and extension of the HA flow to the liver surface.

The main ultrasound findings observed in the 26 patients with BA were gallbladder abnormalities, in all 26 (100%); the TC sign, in 21 (81%); HA enlargement, in 19 (73%); hepatic subcapsular flow, in six (43%); cysts in the porta hepatis, in four (16%); linear or cystic areas within the TC, in three (11%); and polysplenia syndrome, in three (11%). In six patients (43%), even after prolonged fasting, the gallbladder was not visualized during the ultrasound examination (Table 1). Among the 20 patients in whom the gallbladder was visualized, it was classified as atretic in 17 cases (Figure 1). In the remaining three cases, the gallbladder had a longitudinal axis > 1.5 cm with an indistinct mucosal line, an irregular wall, or both (Figure 2). In patients with TC thickening, the TC had a tubular or triangular appearance (Figure 3). Representative examples of HA enlargement and subcapsular arterial flow are shown in Figures 4 and 5, respectively. The cysts in the porta hepatis identified near the right portal vein had thin walls and homogeneous content (Figure 6). In patients in whom the TC sign was detected, hypoechoic or cystic portions of the TC were observed during surgical treatment (Figure 7). Two of the patients with polysplenia syndrome were 56 days old, and the third was 77 days old (Figure 8). Among the 26 patients evaluated, the most relevant secondary findings were hepatomegaly, in 21 (81%); ascites, in 13 (50%); splenomegaly, in nine (35%); and collateral circulation, in seven (27%). The collateral circulation was typically related to portal hypertension and took the form of umbilical vein recanalization.

**Table 1:** Gallbladder characteristics on ultrasound imaging.

Gallbladder characteristic	(N = 26)
Nonvisualized, n (%)	6 (23.1)
Length, n (%)	
≤ 1.5 cm	17 (65.4)
> 1.5 cm	3 (11.5)
Irregular wall, n (%)	
Yes	20 (76.9)
No	0 (0)
Indistinct mucosal line, n (%)	
Yes	16 (61.5)
No	4 (15.4)

RLL, right liver lobe; LLL, left liver lobe; GB, (small) gallbladder; SLL, square liver lobe; GBB, gallbladder bed; C, cystic structure in the porta hepatis; PH, porta hepatis; PVB, portal vein bifurcation; LBHA, left branch of the hepatic artery; RBHA, right branch of the hepatic artery; S, stomach.

## DISCUSSION

The most important ultrasound findings characterizing BA in this series were gallbladder abnormalities, which were present in all patients. In keeping with this finding, the literature shows that the gallbladder is either not visualized or is morphologically altered in most patients with BA^(17^, ^18^, ^19^, ^20)^. In the present study, gallbladder abnormalities were the earliest signs of and the finding most commonly used for the diagnosis of BA. Zhou et al.^(21)^ also reported that as an early finding, as well as showing that it has high specificity and sensitivity for the diagnosis of BA. The gallbladder was not visualized in six patients in our sample; it was visualized and showed morphological changes in the remaining 20 patients, highlighting the importance of adequate pre-examination fasting (for 4–6 h) and the use of a high-frequency linear probe for gallbladder assessment. Our findings are similar to those of Farrant et al.^(22)^, who emphasized the importance of high-frequency linear transducer use in the ultrasound examination of patients with cholestasis, for which they found that nonvisualization or structural alteration of the gallbladder had 90.0% sensitivity, 92.4% specificity, and 91.9% accuracy.

In patients with BA, the appearance of the gallbladder is related to the degree of fibrosis, which can be observed only after the sixth week of life^(16)^. The correlation of ultrasound findings with the surgical classification of BA according to the Japanese Society of Pediatric Surgeons criteria is also important. Subtype III, characterized by fibrosis of the entire biliary tract, with or without gallbladder patency, is the most common form of BA^(16)^. The visualization of a normal gallbladder does not rule out a diagnosis of BA, because the common bile duct, cystic duct, and gallbladder are patent in subtype II^(23)^, which is the rarest form of the disease, accounting for only 2% of cases^(24^, ^25^, ^26)^.

The second most common finding in the present study was the TC sign. Zhou et al.^(21)^ also reported that TC thickening is the second most common ultrasound finding of BA, with a diagnostic sensitivity of 96.9% when considered together with the schematic gallbladder classification. Our findings are similar to those of a subsequent study conducted by Zhou et al.^(14)^, in which changes in the gallbladder and the TC sign were the most common findings (in 100% and 81% of the patients, respectively). In our study sample, TC positivity was defined as a thickness > 2 mm in the most distal portion of the right branch of the portal vein, with or without a thickness > 3 mm anterior to the portal vein bifurcation. No consensus has been reached on those values, given that no reference is made in the relevant literature to the exact points at which the measurements are performed^(7^, ^18)^. The TC sign can be a late finding, appearing after the sixth week of life, because it results from the progression of periportal fibrosis. In some cases, it is necessary to perform a follow-up examination a few weeks after the initial examination, to determine whether the TC sign persists. In a study conducted by Park et al.^(6)^, some patients did not present with hyperechoic TCs early, and it was therefore not possible to use that finding to exclude BA.

In agreement with the literature, HA enlargement (caliber > 1.9 mm) was detected in the majority of cases in the present study. Few authors have described this finding in patients with BA. It appears to be a form of compensation to improve the blood supply to the bile ducts and is commonly observed in patients with liver cirrhosis or vascular malformation. In a review of the literature, Kim et al.^(24)^ found that HA enlargement (defined as an HA caliber > 1.5 mm) showed 92% sensitivity, 87% specificity, and 89% accuracy for the diagnosis of BA, confirming that this finding supports this diagnosis but does not define it in isolation. Some authors, such as Zhou et al.^(14)^, have suggested that HA enlargement cannot be consistently used for the diagnosis of BA, because it can be present in other clinical conditions.

Hepatic subcapsular flow was the only parameter not studied in all of the patients in our sample; it was studied in 14 of the 26 patients because color Doppler assessment was not always possible (in general, the infants were very agitated or constantly crying due to prolonged fasting). Hepatic subcapsular flow has been described in few studies, and variable frequencies of this finding have been reported. Lee et al.^(27)^ observed it in all of their patients. In another, previous, study conducted by Lee et al.^(26)^, all patients with BA who had hepatic subcapsular flow at the time of the Kasai procedure had telangiectatic vessels on the liver surface. Zhou et al.^(14)^ reported that hepatic subcapsular flow should not be used in isolation for the diagnosis of BA, because its detection depends greatly on the ultrasound device used and the appropriate adaptation of the color Doppler parameters. These findings align with ours, given that the presence of hepatic subcapsular flow did not correlate well with the diagnosis of BA in our study.

Porta hepatis macrocysts or microcysts were detected infrequently in our series. That finding was recently described as important for the diagnosis of BA^(24)^. Koob et al.^(28)^ found that the presence of a microcyst alone had a specificity close to 98% for the diagnosis of the disease. These cysts occur in the cystic form of BA; they appear in the second trimester of pregnancy and can thus be detected by ultrasound during the fetal period^(24)^. Cysts in the porta hepatitis should not be confused with choledochal cysts.

Distinct TCs with linear, tubular, or round hypoechoic or cystic portions representing patent segments of the fetal bile duct were present in 11% of our patients. We found only one study detailing the presence and prevalence of these remnants; Kim et al.^(24)^ correlated the presence of these TC structures, visualized by ultrasound or magnetic resonance cholangiography, with histopathological findings, showing that they are useful in the differential diagnosis of BA. We found that patients with these fetal remnants who could undergo the Kasai procedure had better prognoses. The last patient in our sample had a patent remnant in the porta hepatis, detected by ultrasound and macroscopically during porta hepatis dissection at the age of 195 days; the Kasai procedure was performed, with a very favorable outcome, characterized by improved liver function. To our knowledge, the presence of these fetal remnants has not been correlated with the best outcome for this patient population.

Polysplenia syndrome is rare and is the structural malformation most frequently associated with the embryonic form of BA. In our sample, one patient presented with situs inversus and polysplenia, one presented with a preduodenal portal vein and polysplenia, and one presented with situs inversus alone. Koob et al.^(28)^ equated the presence of polysplenia syndrome with other ultrasound findings of BA, reporting that it had a high degree of diagnostic specificity.

Secondary findings are nonspecific for the diagnosis of BA. Most such findings are consequences of the progression to cirrhosis and portal hypertension for distinct reasons, including the natural course and often late diagnosis of the disease. The patients in our sample with hepatomegaly, splenomegaly, ascites, and collateral circulation were diagnosed later with BA. Humphrey et al.^(25)^ described hepatosplenomegaly as a weak predictor of BA.

Ultrasound is useful for the exclusion of BA, but it is limited by the degree of operator experience. Although the feasibility of magnetic resonance cholangiography for the reliable diagnosis of BA has been examined, its reported diagnostic value, in particular its specificity, varies widely^(7^, ^21)^: 90–100% sensitivity; 36–96% specificity; and 71–98% accuracy.

This study has some limitations. First, it had a cross-sectional retrospective design. In addition, gallbladder abnormalities were assessed subjectively, with the interpretation of the findings depending on examiner experience. Finally, the small dimensions of the structures examined, such as the TC and HA, could have resulted in measurement errors.

## CONCLUSION

In conclusion, with the consideration of TC parameters and the use of a gallbladder classification that is more objective, neonatal BA can be identified by ultrasound. Cysts in the portal hepatis are important abdominal ultrasound findings in patients with neonatal cholestasis. Finally, as new data indicate, fetal remnants visualized in the ultrasound and confirmed during surgical exploration should be described in the ultrasound, because this technique confers a better prognosis when it is possible to perform the Kasai procedure.
